# High Bactericidal Self-Assembled Nano-Monolayer of Silver Sulfadiazine on Hydroxylated Material Surfaces

**DOI:** 10.3390/ma12172761

**Published:** 2019-08-28

**Authors:** Angelo Taglietti, Giacomo Dacarro, Daniele Barbieri, Lucia Cucca, Pietro Grisoli, Maddalena Patrini, Carla Renata Arciola, Piersandro Pallavicini

**Affiliations:** 1 Dipartimento di Chimica, Sezione di Chimica Generale, Università di Pavia, viale Taramelli 12, 27100 Pavia, Italy; 2 Dipartimento di Scienze del Farmaco, Università di Pavia, viale Taramelli 10, 27100 Pavia, Italy; 3 Dipartimento di Fisica, “A. Volta”, Università di Pavia, via Bassi 6, 27100 Pavia, Italy; 4 Laboratorio di Patologia delle Infezioni Associate all’Impianto, IRCCS Istituto Ortopedico Rizzoli, via di Barbiano 1/10, 40136 Bologna, Italy; 5 Department of Experimental, Diagnostic and Specialty Medicine, University of Bologna, via San Giacomo 14, 40126 Bologna, Italy

**Keywords:** antimicrobial, nanocoatings, self-assembled monolayer, anti-infective materials, sulfadiazine

## Abstract

Anti-infective surfaces are a modern strategy to address the issue of infection related to the clinical use of materials for implants and medical devices. Nanocoatings, with their high surface/mass ratio, lend themselves to being mono-layered on the material surfaces to release antibacterial molecules and prevent bacterial adhesion. Here, a “layer-by-layer” (LbL) approach to achieve a self-assembled monolayer (SAM) with high microbicidal effect on hydroxylated surfaces is presented, exploiting the reaction between a monolayer of thiolic functions on glass/quartz surfaces and a newly synthesized derivative of the well-known antibacterial compound silver sulfadiazine. Using several different techniques, it is demonstrated that a nano-monolayer of silver sulfadiazine is formed on the surfaces. The surface-functionalized materials showed efficient bactericidal effect against both Gram-positive and Gram-negative bacteria. Interestingly, bactericidal self-assembled nano-monolayers of silver sulfadiazine could be achieved on a large variety of materials by simply pre-depositing glass-like SiO_2_ films on their surfaces.

## 1. Introduction

Bacterial infections associated with the use of medical devices are a heavy worldwide health issue, with serious clinical repercussions involving an enormous number of people [1]. These infections commonly result from the irreversible adhesion of bacteria to the device surfaces, often leading to biofilm growth [2], and remarkably resist both the immune defenses and systemic antibiotic therapies. As they are usually inherently protected from antibacterial natural and medical weapons, it’s a good strategy to prevent bacteria growth from the beginning. The ideal goal is to impede or render impossible bacterial adhesion to material surfaces. The first choice should be to modify medical device inorganic surfaces with coatings of antibacterial agents, in order to limit bacteria presence in the surrounding of surfaces [3].

The self-assembled monolayers (SAM) approach is a straightforward tool for the surface modification [4] and SAMs (self-assembled monolayers) deposited on silicon or gold substrates have been used as model surfaces to study the adhesion of bacteria as influenced by the surface features [5,6,7].

Covalent bonding on various surfaces of different antibacterial functions has been described [8,9,10,11,12,13,14,15,16]: Quaternary ammonium groups [8] and antibiotic, such as vancomycin [9], are straightforward examples from the last decades. A self-assembled monolayer (SAM) of -COOH terminated phosphonic acid has been deposed on hydroxyapatite, and subsequently loaded with silver ions, yielding a material with some antibacterial effect [10]. Using a similar approach, a self-assembled phosphonate monolayer functionalized with silver thiolate species was realized on metal surfaces, showing antibacterial properties [11]. Copolymers covalently bound to different fibers and surfaces [15] and carbon nanotubes bound to polyamide membranes [16] were also used. A very popular approach is the one which exploits the “layer-by-layer” (LbL) deposition technique [17,18,19] applied to graft silver nanoparticles (Ag NP) on SAMs capable of interaction with metallic silver [20,21,22]. Other interesting examples of the use of the SAM technique have been recently proposed [23,24,25,26,27].

Moreover, even more recently, silver nanoparticles have been adopted to open up new-avenues for biocompatible nano-medicines. In this connection, ecofriendly technologies are emerging as potential anti-infective strategies in searching for alternatives to conventional antibiotics [28,29,30,31,32,33]. Ag^+^ is an environmentally friendly antimicrobial, as (i) it is highly toxic for a wide range of micro-organisms [34], (ii) it is non-toxic (in low concentrations) for human cells, (iii) bacteria are not able to develop resistance, as Ag^+^ attacks a broad range of targets in organisms, and thus, resistance should involve several simultaneous mutations [35]. This allows to foresee its use as a powerful antibiotic for the future [36,37,38,39,40,41,42,43], with a particular interest for its use in antibacterial coatings [38,39,40,41,42,43,44,45].

**Scheme 1:**
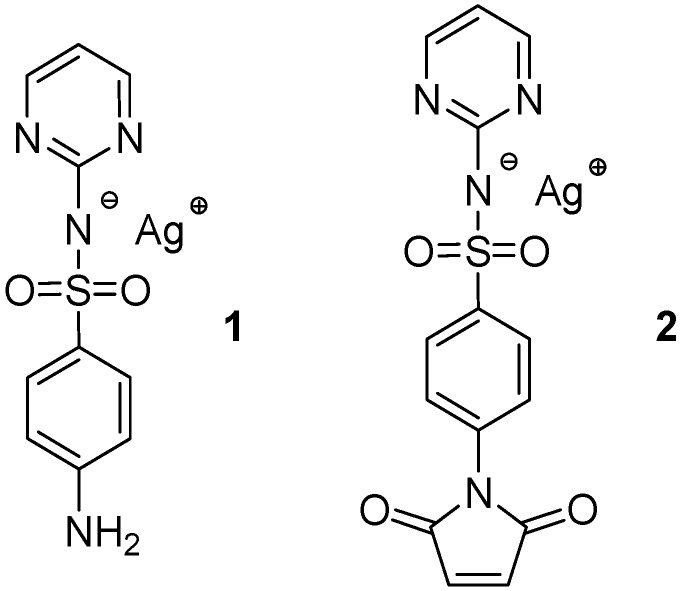
Silver sulfadiazine (**1**) and silver sulfadiazine maleimide (**2**).

Silver sulfadiazine (Ag-SD, 1, see Scheme 1) is a very powerful Ag^+^ based antimicrobial agent. It possesses a well-known antibacterial activity [46] and also strong antifungal properties [47] and is a clinically used topical agent for treatment of burn wound infections [48,49]. Sulfadiazine itself does not show any antibacterial activity, which is observed only in synergic presence of Ag^+^ and is related to slow release of Ag^+^ from the poorly insoluble sulfadiazine salt [45].

**Scheme 2:**
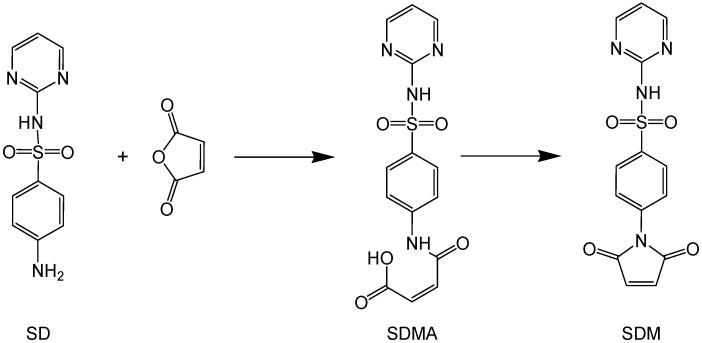
Synthesis of sulfadiazine maleimide (SDM).

In this context, with the aim to impart to inorganic surfaces an intrinsic antibacterial action based on Ag^+^, we used the LbL approach to coat flat glass or quartz slides with an Ag-SD derived fragment, obtained with a new, expressly designed synthesis, and verified the antibacterial activity of these modified surfaces [44]. As far as we know, this is the first report describing a method to covalently bind a very active antibacterial Ag-SD derived fragment to surfaces. We chose glass, as a representative economic material, but the new method proposed here can be applied to any hydroxylated surface, such as paper or cotton fibers. The LbL approach resembles the one we used for preparation of silver NP monolayers: First, a SAM of thiols (using a mercaptosilane reagent) was assembled on activated glass, following a procedure that was optimized in our laboratories [44]. Then, the -SH coated glass was immersed in a solution containing the silver salt of sulfadiazine maleimide (Ag-SDM, see Scheme 2): transformation of aromatic amine in a maleimide was chosen as the coupling reaction between an -SH terminated SAM function and maleimide is very efficient [50].

## 2. Results

### 2.1. Synthesis and Characterization

**Scheme 3:**
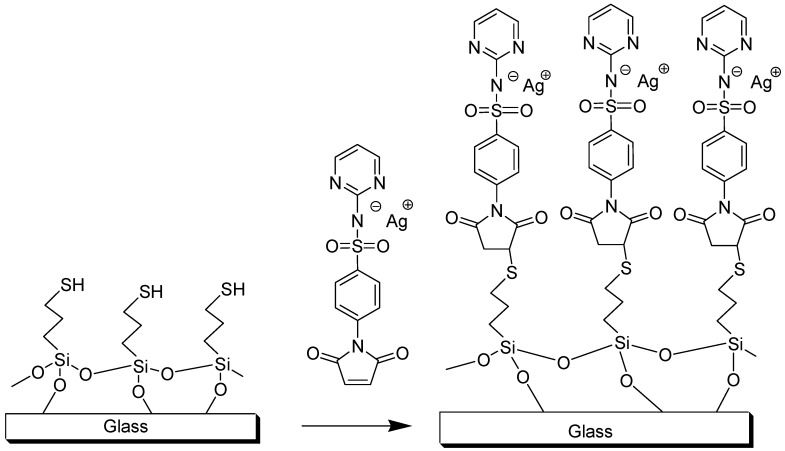
Synthesis of SURF -Ag-SDM glasses.

**Figure 1:**
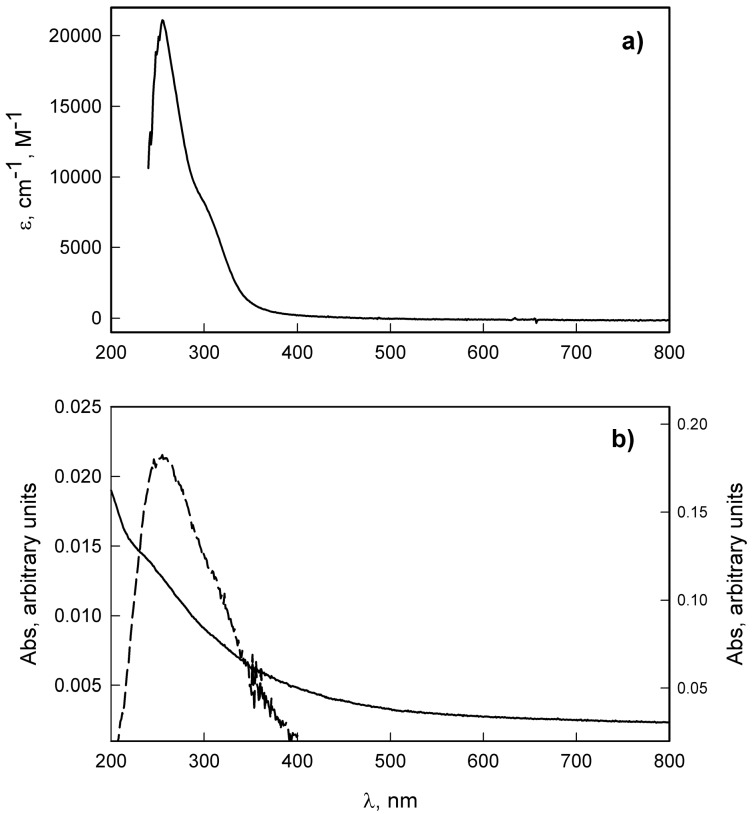
(**a**) UV-Vis spectrum of a 10^−4^ M solution of Ag-SDM in water/DMSO in a quartz cuvette, (**b**) UV-Vis spectrum of SURF-Ag-SDM i.e., a quartz slide coated with a SAM of Ag-SDM (solid line, absorbance on the right axis). The dashed spectrum (absorbance on the left axis) is obtained after background subtraction (the scale of the axis is smaller), in order to evidence the absorption band.

The reaction between thiols on the surface and Ag-SDM 2 leads to the self-assembly of a monolayer covalently attached to the modified glass, as sketched in Scheme 3. The typical preparation of modified glasses involves reaction of at least eight samples of -SH terminated glasses with Ag-SDM. Grafting of Ag-SDM was obtained by dipping the -SH terminated surfaces in a freshly prepared water/DMSO solution of Ag-SDM for 4 h at room temperature. Four contact angle measurements were performed on each sample, before and after immersion. Static contact angle changes from the value of 63° (±3°) for the SH terminated surface to a value of 46° (±3°) after functionalization with Ag-SDM. UV-Vis spectra were recorded on quartz surfaces functionalized with the Ag-SDM SAM (prepared with the same procedure used for glass) and corrected by background subtraction [50,51]. The obtained spectra of Ag-SDM functionalized quartz, as reported in Figure 1b, shows an UV band centered at 255 nm, which is the same band observed for Ag-SDM in solution (λ_max_ = 255 nm ε = 21,000 cm^−1^ M^−1^, Figure 1b). From the absorbance value at λ_max_ (A), by using the molar extinction coefficient found in solution, (ε) and the relation n_s_(cm^−2^) = 6 × 10^20^ A/2ε, the number of cromophores per square centimeter can be calculated [48]: n_s_ = 3.0 (0.5) 10^14^ cm^−2^ (average on three samples). The value is in agreement with the values already found in similar coating procedures [50,51,52,53]. The Ag^+^/cm^2^ concentration in the SURF-Ag-SDM SAM was measured directly by de-metallating four coated glass slides dipping them in 0.5 M HNO_3_ (each glass slide in a 3 mL volume, 24 h) and measuring the total silver content in the obtained solution with inductively coupled plasma atomic spectroscopy (ICP-OES). We found an average value of 2.4 (0.4) 10^14^ silver atoms/cm^2^. This value obviously corresponds to the number of Ag-SDM molecules which reacted with thiol functions on glass, i.e., the surface density of Ag-SDM on glass after reaction with the thiols. Once again, this value is in very good agreement with those already found in literature [50,51,52,53] and with the data obtained from UV spectra, and strongly suggests the formation of a monolayer of Ag-SDM covalently bound to thiols on glass. UV-Vis spectra on Ag-SDM functionalized quartz, ICP-OES determination of total quantity of silver ions bound on surfaces in SURF-Ag-SDM glass, and contact angle measurements, when repeated on aged (up to two months) samples, always gave the same results found on the fresh samples. This indicates the stability of the samples within this timescale. Thus, samples can be stored in the dark, without any other precaution, and used within two months from the preparation.

In order to be able to test the antibacterial efficiency of sulfadiazine in absence of Ag^+^, eight -SH terminated glasses were dipped in an SDM solution, i.e., sulfadiazine maleimide in absence of silver ion. In this case, after the grafting (samples SURF-SDM), the static contact angle changed to a value of 51° (±3°).

FTIR-ATR spectroscopy and spectroscopic ellipsometry confirmed the nature of the SURF-SDM and SURF-Ag-SDM SAMs. FTIR-ATR spectra were measured on SURF-SH, SURF-SDM and SURF-AgSDM SAMs.

**Figure 2:**
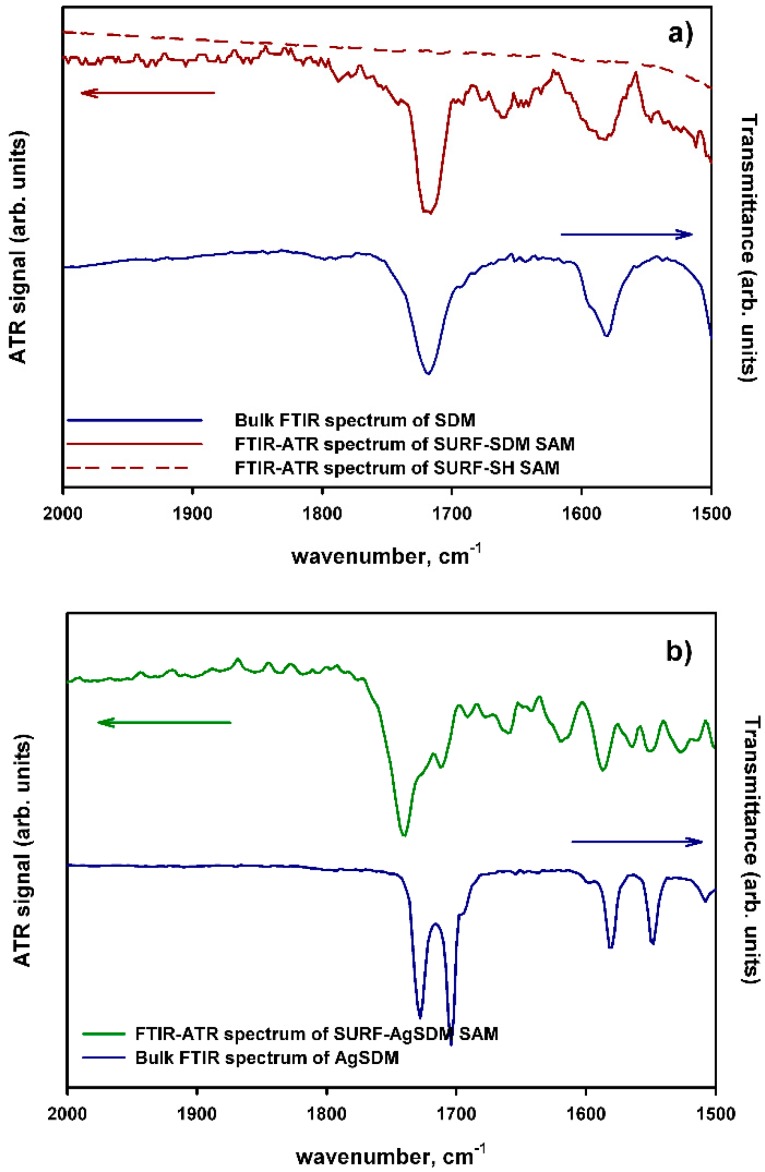
FTIR ATR spectra of (**a**) SURF-SDM (solid red line) SURF-SH (dashed red line) and bulk SDM (solid blue line); (**b**) SURF-Ag-SDM (green line) and bulk Ag-SDM (solid blue line).

As can be clearly seen in Figure 2, FTIR-ATR bands in the 1500–2000 cm^−1^ region are comparable to those found on bulk SDM and Ag-SDM. SURF-SDM (red plot) and SURF-AgSDM (green plot) show the same features of the corresponding bulk spectra (blue plots) measured in transmission FTIR with nujol mull and NaCl windows. SURF-SH SAM (red dashed plot) spectrum has no significant features in this spectral region. Ellipsometric spectra on SURF-SH, SURF-SDM and SURF-AgSDM were measured from 250 to 900 nm on samples prepared on the silicon crystal substrates used for ATR measurements, with a procedure identical to that applied to the glass slides (see Figure S1).

Optical functions for the SDM and AgSDM monolayers have been modeled with a Sellmeier-type behavior for the refractive index n (considering the value of 1.679 reported in literature for plain sulfadiazine) joined to gaussian-like extinction coefficient k associated to the UV absorption band, centered at 255 nm. The functionalization with SDM and AgSDM produces an effective overlayer thickness of 1.09 and 1.05 nm, respectively. These values are in good agreement with the calculated molecule length of 1.3 nm (obtained from PM3 molecular modeling with Hyperchem™ 6.03 package, see Figure S2), indicating the formation of single molecular layers with a thickness of 1.09 nm and 1.05 nm for SURF-SDM and SURF-Ag-SDM, respectively. The combination of the results FTIR-ATR, ellipsometry, UV-Vis spectra and Ag surface concentration allows to state that a monolayer of AgSDM is formed on thiolated glasses and quartz, as a result of the efficient coupling reaction between -SH and maleimide.

### 2.2. Antibacterial Tests

Antibacterial activity tests were performed on three sets of differently coated samples, in order to evaluate the different contributions from different antibacterial species (Ag^+^ and SDM) involved in the coating: SURF-SH, SURF-SDM and SURF-Ag-SDM.

In order to evaluate the efficiency of these materials in preventing the local growth of bacteria in a coated surface surrounding, we developed a test procedure in our laboratory [44] which is capable of measuring the microbicidal effect [54]. This test allows the simulation of real-life conditions of use, i.e., the evaluation of the bactericidal effect in a thin liquid film in contact with the coated surface. *Stapylococcus aureus* (ATCC *6538*) and *Escherichia coli* (ATCC *10356*) were used as commonly considered representative bacteria types, and the results were compared with those obtained for the same strains using SAM of silver NP grafted on glass in different ways [44,51].

ME for the samples prepared in this work are summarized in Table 1. In the test proposed by EN 13967 [54], the microbicidal activity is considered efficient when ME is at least equal to 4, a value which corresponds to elimination of 99.99% of bacteria placed in contact to the surface. As reported in Table 1, we measured the ME values after 5 and 24 h of contact, focusing on materials exerting long-lasting antibacterial effects. ME is absent for glasses functionalized with thiols and sulfadiazine. On the contrary, the SURF-Ag-SDM glass slides showed a very efficient ME, increasing enormously moving from 5 to 24 h of contact with the glasses functionalized with silver sulfadiazine. It is worth noting that this ME is obtained with an amount of silver which is very small, especially when compared (<15%) to other surfaces showing comparable ME that we previously described [44,51]. To have a rough evaluation of the quantity of Ag^+^ released during the contact time, we aged the functionalized glass slides SURF-Ag-SDM (eight samples) for 5, 24, and 48 h in bidistilled water (pH 7.5, each glass slide in a 3 mL volume) and then measured Ag^+^ released with ICP-OES. Ag^+^ found in all samples was close to the detection limit (10 ppb), and the average value found was of 20 (±10) ppb for all of the three dipping times, corresponding to 5.6 × 10^−10^ mol of Ag^+^ released from a glass slide in 3 mL of water. Moreover, UV-Vis spectra of the aqueous solutions were collected, in order to verify the presence of Ag-SDM complex absorption band at 255 nm. No absorption band was detected.

## 3. Discussion

A crucial aspect in the pathogenesis of device-related infection is the ability of bacteria to produce biofilms, communities of bacterial cells embedded in extracellular polymeric matrixes [55]. Biofilm formation starts when bacteria adhere to the material surface [56]. Once the biofilm structure has developed and matured, some bacteria detach and spread at distance over the biomaterial surface, thus, propagating infection and perpetuating biofilm formation [2,55]. Therefore, strategies able to prevent the initial bacterial adhesion on the material surface may hamper biofilm formation and infection onset [2,3]. In this connection, the new nanocoating here presented aims precisely to combat implant-related infection from the beginning.

The data obtained from UV spectra strongly suggest the formation of a monolayer of Ag-SDM covalently bound to thiols on glass.

This monolayer bears a small amount of silver, not enough to exert toxic effects on eukaryotic cells, and grants a permanent binding to the material surface, thus ensuring both stability of the antimicrobial coating and biological safety. Indeed, the firm anchorage of a simple monolayer of silver containing complex via covalent bound abolishes the risk of toxic effects for immune cells that can derive from freely circulating detached silver complexes or nanoparticles [57].

Moreover, the absence of an absorption band at 255 nm in solution after prolonged immersion of samples in water is a finding that allows us to reasonably exclude that silver is released as Ag-SDM complex in an appreciable quantity. Comparing the quantity of silver released from a slide in 3 mL of water after immersion times in the range from 5 to 48 h (5.6 × 10^−10^ mol of Ag^+^) with the total quantity of silver brought on a slide detected from ICP measurements (2.4 (0.4) × 10^14^ silver atoms/cm^2^, corresponding to 28 (5) × 10^14^ silver atoms /slide, corresponding to 4.7 (0.8) × 10^−9^ mol of Ag^+^ on a single slide), it is possible to calculatethat less than 12% of the total silver present on the surface is released as Ag+ ion from the glass slides when immersed in large (as compared to those used in the assay to evaluate the microbicidal effect) volumes of distilled water up to 48 h. The quantity of silver released in the environment in these conditions is very low, when compared with already cited surfaces coated with a monolayer of silver NP [44,51]. These data also suggest that the silver-sulfonamido groups are stable toward hydrolysis. Moreover, it seems reasonable that silver release during the microbicidal effect test, which involves smaller volumes of highly saline liquid, should be even lower than that measured in water. It is interesting to notice that silver ions seem to be released mainly in the first hours of contact with water, as the concentration values found after immersion of SURF-Ag-SDM samples in water solution do not change appreciably moving from 5 to 48 h. On the other hand, the microbicidal effect strongly increases moving from 5 to 24 h of contact. These data suggest that the microbicidal action is connected with accumulation inside bacteria of silver ions which are released from functionalized glass. It seems reasonable that Ag^+^ is up-taken by free thiols groups exposed at the surface of the bacterial membrane proteins [58] upon contact with the silver sulfadiazine groups grafted on the surface of the glass slides or upon draining from the aqueous solution which is in contact with the slides. Moreover, the fact that a strong ME is observed only in presence of Ag^+^ demonstrates that sulfadiazine, as expected, is not able to exert its antibacterial activity but plays an essential role in the mechanism of action of its silver salt. Thus, the efficient ME of SURF-Ag-SDM is to be ascribed to “controlled” release of Ag^+^ from the sulfadiazine salt covalently bound to the surface.

## 4. Materials and Methods

*Materials.* Reagents and solvents were purchased from Sigma-Aldrich (St. Louis, MO, USA) and used as supplied. Microscopy cover glass slides (2.4 × 2.4 cm) were purchased from Forlab (Carlo Erba, Milan, Italy). Quartz slides (Spectrosil 2000 fused silica, 1.4 × 1.4 cm, 1 mm thick) were purchased from UQGOptics Ltd. Water was bidistilled, prepared from deionized samples.

*Synthesis of SDMA.* Sulfadiazine, SD (10 g, 0.04 mol) and maleic anhydride (0.05 mol) were solved in 75 mL dimethylsulfoxide. After 3 h, 75 mL water were added to the reaction mixture and a pale yellow precipitate formed. The solid product was collected by filtration, washed with a small quantity of DMSO:H_2_O mixture (1:1), washed with water and dried at 40 °C. Sulfadiazine-maleammic acid (SDMA) is obtained in quantitative yield.

MS(ESI): 349 *m*/*z* [M + H]^+^

1H NMR (DMSO): δ = 6.32 and 6.45 (d and d, 1H each, CH=CH), δ = 7.04 (t, 1H, pyrimidine H, para), δ = 7.78 e δ = 7.94 (d and d, 4H, phenylic protons), δ = 8.50 (d, 2H, pyrimidine H, meta), δ = 10.7 (s, 1H, SO_2_NH), δ = 11.7 (very broad, COOH/NHC=O). See Figure S3.

IR (*nujol*): 1131.96 and 1336.40 cm^−1^ (stretching S=O), 1319.35 cm^−1^ (stretching C-N secondary aromatic amine), 1581.86 cm^−1^ (aromatic C-H), 1631.33 cm^−1^ (bending aromatic amine), 1721.72 cm^−1^ (stretching C=O).

*Synthesis of SDM.* Sulfadiazine-maleammic acid (SDMA) (13.6 g, 0.07 mol) was solved in 120 mL acetic anhydride. Anhydrous sodium acetate (1.6 g, 0.5eq) was added and the mixture was heated at 80 °C for 15 min. A brown precipitate formed. Reaction mixture was cooled at room temperature and 250 mL of ice and water mixture was added. Five extractions with 50 mL CHCl_3_ were performed, organic phases were collected and dried over sodium sulfate. Chloroform solution was filtered and evaporated, obtaining a thick yellow oil. Crude product was repeatedly washed with heptane to remove traces of acetic acid. The oil was then dried in high vacuum and the product was further purified with a recrystallization in chloroform/hexane, obtaining a white solid. (yield: 81%)

MS(ESI): 331 *m*/*z* [M + 1]^+^

1H NMR (DMSO): δ = 7.05 (t, 1H, pyrimidine H, para), δ = 7.23 (s, 2H, maleimidic CH=CH), δ = 7.60 e δ = 8.09 (d e d, 4H, phenylic protons), δ = 8.53(d, 2H, pyrimidine H, meta), δ = 11.9 (broad s, SO_2_NH). See Figure S4.

IR (nujol): 1159.23 and 1345.73 cm^−1^ (stretching S=O), 1500.47 and 1578.78 cm^−1^ (aromatic C-H), 1722.73 cm^−1^ (stretching C=O)

*Synthesis of AgSDM.* A boiling solution of SDM in ethanol (200 mL, 2 × 10^−3^ M) was added under stirring to a boiling silver nitrate solution in ethanol (200 mL, 2 × 10^−3^ M). A white precipitate formed cooling the solution to room temperature. The solid was isolated by centrifugation and dried under high vacuum (yield: 83%).

^1^H NMR (DMSO): δ = 6.88 (t, 1H, pyrimidine H, para), δ = 7.21 (s, 2H, maleimidic CH=CH), δ = 7.48 e δ = 8.11 (d and d, 4H, phenylic protons), δ = 8.48 (d, 2H, pyrimidine H, meta). See Figure S4.

IR (*nujol*): 1146.65 and 1417.22 cm^−1^ (stretching S=O), 1580.26 and 1549.04 cm^−1^ (aromatic C-H), 1701.67 and 1729.42 cm^−1^ (stretching C=O).

*Preparation of (3-mercaptopropyl)trimethoxysilane SAM (SURF-SH).* Cover glass slides were cleaned with piranha solution and then washed three times with ultrapure water under sonication. Glasses were then immersed for 4 h in a 5% (*v/v*) solution of (3-mercaptopropyl)trimethoxysilane (MPTS) in toluene and kept thermostatted at 40 °C. After this, the thiol modified glasses were washed under sonication with a sequence of toluene, then a mixture (50% *v*/*v*) of toluene and ethanol, and then ethanol. Finally, they were dried under a nitrogen stream (50).

*Preparation of sulfadiazine SAM (SURF-SDM).* SURF-SH glasses were immersed for 4 h in a 10^−4^ M SDM solution in PBS (pH 7) and kept thermostatted at 30 °C. The sulfadiazine modified glasses were then washed under sonication with ultrapure water and ethanol and finally dried under a nitrogen stream.

*Preparation of silver sulfadiazine SAM (SURF-AgSDM).* SURF-SH glasses were immersed for 4 h in a 10^−4^ M AgSDM solution in DMSO: aqueous phosphate buffer mixture (9:1, buffered at pH 7) and kept thermostated at 30 °C. The silver sulfadiazine modified glasses were then washed under sonication with a sequence of DMSO, ultrapure water and ethanol. The glasses were finally dried under a nitrogen stream.

*Antibacterial activity tests.* The antibacterial activity of SURF-SDM and of SURF-AgSDM and of SURF-SH monolayers was investigated as in [23]. Briefly, the test materials were challenged with *Staphylococcus aureus* ATCC 6538 (Gram+) and *Escherichia coli* ATCC 10356 (Gram-). Bacteria from overnight broth cultures were used to prepare 10^8^ CFU (colony forming unit) suspensions in Dulbecco’s PBS. Bacterial suspensions (10 µL) were placed on conventional glass slides (26 × 76 mm) and covered with the test nano-coated glass slides (24 mm × 24 mm). This two-glasses system was maintained in damp milieu (with 1 mL of PBS into a Falcon test-tube) at room temperature for 5 and 24 h. The same setup was used with an untreated glass slide as a control. After the two indicated contact times, a volume of 9 mL of PBS was added in each test-tube, gently shaking in order to obtain the detachment of the two glass slides. Bacterial suspensions were then placed in Tryptone Soya Agar (Oxoid; Basingstoke, Hampshire, England) and grown, in order to count viable cells. The decimal-log reduction rate, microbicidal effect (ME), was calculated using the formula: ME = log NC − log NE (NC being the number of CFU/mLcounted in the case on the untreated control glasses, and NE being the number of CFU/mLcounted after exposure to coated glasses). The results expressed as ME are the average of three equivalent determinations.

*FTIR-ATR characterization of SURF-SDM and SURF-AgSDM functionalized surfaces* FTIR-ATR (Fourier transform infrared-attenuated total refelectance) spectra were measured on SURF-SH, SURF-SDM and SURF-AgSDM SAMs by a Bruker IFS113v spectrometer, equipped with a home-made setup for multiple reflection ATR. Samples were prepared directly on double-polished silicon ATR crystals (Korth Kristalle Gmbh, 10 mm × 50 mm × 3 mm, trapezoidal shape with 45° bevels). Each spectrum is collected with 256 scans at a spectral resolution of 4 cm^−1^. The sample compartment is kept under vacuum (10^−3^ torr).

*Spectroscopic Ellipsometry* Ellipsometric spectra on SURF-SH, SURF-SDM and SURF-AgSDM were measured from 250 to 900 nm with an SOPRA ES4G rotating polarizer ellipsometer, equipped with a single-photoncounting photomultiplier detector, at angles of incidence of 70 and 75 degrees. Experimental results were analyzed with the WVASE32 software from J.A. Woollam Inc.

Samples were prepared on the same silicon crystals used for ATR measurements, with a procedure identical to that applied to the glass slides.

Reference measurements on the different silicon crystals before functionalization allow to determine native SiO_2_ oxide thickness. Silanization with MPTS produces an effective overlayer whose average thickness is determined adopting SiO_2_ optical functions [47]. A total effective thickness of 4.09 and 3.70 nm was measured on the two samples.

Optical functions for the SDM and AgSDM monolayers have been modelled with a Sellmeier-type behavior for the refractive index n (considering the value of 1.679 reported in literature for plain sulfadiazine) joined to gaussian-like extinction coefficient k associated to the UV absorption band, centered at 255 nm.

*UV-Vis spectroscopy.* Absorbance spectra of functionalized glasses or quartz were taken with a Varian Cary 100 spectrophotometer equipped with a dedicated Varian solid sample holder. Absorbance spectra of solutions were taken with the same instrument equipped with a standard cuvette holder.

*Inductively coupled plasma atomic emission spectroscopy.* Inductively coupled plasma optical emission spectroscopy (ICP-OES) data were collected with an ICP-OES OPTIMA 3000 Perkin Elmer instrument. The total Ag content on SURF-AgSDM glasses was determined by quantitatively oxidizing/demetallating the samples (24 mm × 24 mm or 12 mm × 24 mm) by deeping them in 5mL ultrapure concentrated HNO_3_ diluted 1:5 with water (13% was final concentration) in a vial, and keeping it at RT on a Heidolph Promax 1020 reciprocating platform shaker overnight. The Ag^+^ release from glass samples in water was obtained dipping SURF-AgSDM samples in 3 mL of bidistilled water for 5, 24, and 48 h (eight samples for each time interval). The Ag content in solution was then determined by ICP-OES.

*Mass spectroscopy and NMR.* Mass spectra (electrospray ionization–ionic trap) were recorded on a Thermo Finnigan LCQ Advantage Max, NMR spectra (400 MHz) on a Bruker AMX 400 spetrometer.

## 5. Conclusions

In conclusion, we prepared glass and quartz samples functionalized with a well-established antibacterial agent, silver sulfadiazine, by means of simple and reproducible synthetic steps. Moreover, we demonstrated the existence of a SAM using different techniques which allowed us to measure (i) the total quantity of Ag-SDM brought to quartz surfaces (by UV-Vis spectroscopy), found compatible with the formation of a monolayer, (ii) the total quantity of silver present on glass samples, obtained via acid digestion of glasses followed by ICP-OES on acid solutions (once again compatible with a monolayer presence), (iii) the layer thickness, obtained by ellipsometry, consistent with the presence of a nano-monolayer, (iv) FTIR ATR spectra, confirming that the monolayer is composed of Ag-SDM.

To our knowledge, this is the first report on the efficient microbicidal action of a classical antibacterial agent such as silver sulfadiazine covalently bound to an inexpensive surface by means of a very simple synthetic technique, a result which opens an important route to realization of implants, medical devices (provided with any generic hydroxylated or -SH coated surface) inherently protected from bacteria and biofilm growth.

## Figures and Tables

**Table 1:** Microbicidal effect (ME) values ^a^.

	*S. aureus*		*E. coli*	
	5 h	24 h	5 h	24 h
SURF-SH	−0.1	−0.1	−0.2	−0.3
SURF-SDM	0.4	0.2	0.3	0.1
SURF-Ag-SDM	0.8	6.1	1.0	4.0

^a^ All values are obtained as an average of four experiments. ME = log N_C_ − log N_E_ (N_C_ is the number of CFU/mLdeveloped on the unmodified control glasses, and N_E_ being the number of CFU/mLcounted after exposure to modified glasses; CFU = colony forming unit).

## Supplementary Materials

The following are available online at https://www.mdpi.com/1996-1944/12/17/2761/s1, Figure S1. Spectroscopic Ellipsometry. Figure S2. Sulfadiazine maleimide (SDM) PM3 model. Figure S3. NMR spectra of SDMA (with enlarged zone of characteristic protons). Figure S4. NMR Spectra for SDM and AgSDM (with enlarged zone of characteristic protons).
