# Supplementary material for: High Bactericidal Self-Assembled Nano-Monolayer of Silver Sulfadiazine on Hydroxylated Material Surfaces
Source: Materials (Basel). 2019 Aug 28;12(17):2761. doi: 10.3390/ma12172761 (PMC6748069; doi:10.3390/ma12172761)
Supplement: Supplementary file 1 [file materials-12-02761-s001.pdf]

materials

Supplementary Materials

High Bactericidal Self-Assembled Nano-Monolayer of Silver Sulfadiazine on Hydroxylated Material Surfaces

Angelo Taglietti [1,*], Giacomo Dacarro [1,*], Daniele Barbieri [1], Lucia Cucca [1], Pietro Grisoli [2], Maddalena Patrini [3], Carla Renata Arciola [4,5,*] and Piersandro Pallavicini [1]

[1] Dipartimento di Chimica, Sezione di Chimica Generale, Università di Pavia, viale Taramelli 12, 27100 Pavia, Italy
[2] Dipartimento di Scienze del Farmaco, Università di Pavia, viale Taramelli 10, 27100 Pavia, Italy
[3] Dipartimento di Fisica, "A. Volta", Università di Pavia, via Bassi 6, 27100 Pavia, Italy
[4] Laboratorio di Patologia delle Infezioni Associate all'Impianto, IRCCS Istituto Ortopedico Rizzoli, via di Barbiano 1/10, 40136 Bologna, Italy
[5] Department of Experimental, Diagnostic and Specialty Medicine, University of Bologna, via San Giacomo 14, 40126 Bologna, Italy
* Correspondence:angelo.taglietti@unipv.it (A.T.); giacomo.dacarro@unipv.it (G.D.);carlarenata.arciola@ior.it (C.R.A.)

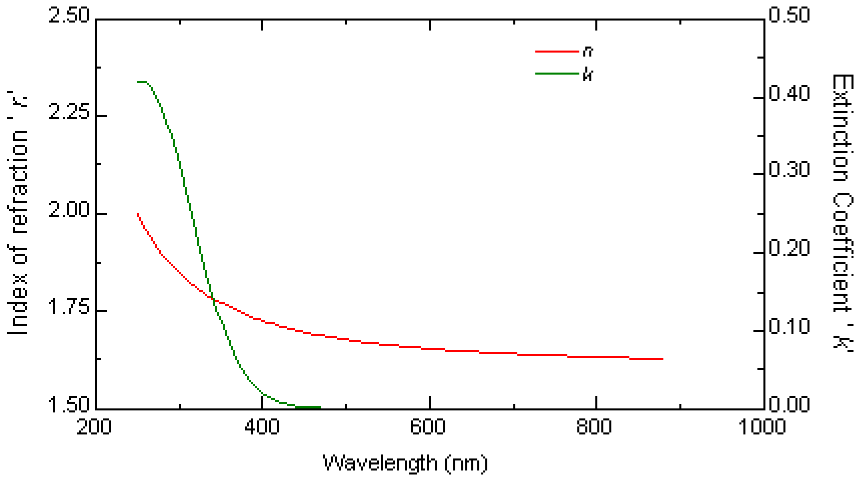

Figure S1. Spectroscopic Ellipsometry.

Optical functions for the SDM and AgSDM monolayers have been modeled with a Sellmeier-type behavior for the refractive index n (considering the value of 1.679 reported in the literature for plain sulfadiazine) joined to gaussian-like extinction coefficient k associated to the UV absorption band, centered at 255 nm.

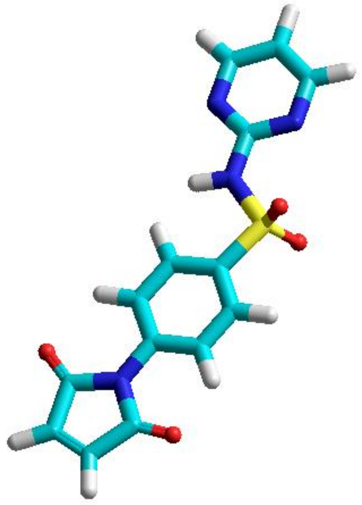

Figure S2. Sulfadiazine maleimide (SDM) PM3 model.

SDM molecular model obtained with a semi-empirical PM3 method. In its extended conformation SDM has a length of 1.3 nm (distance between a C atom of the maleimidic double bond and the terminal C atom of the pyrim.

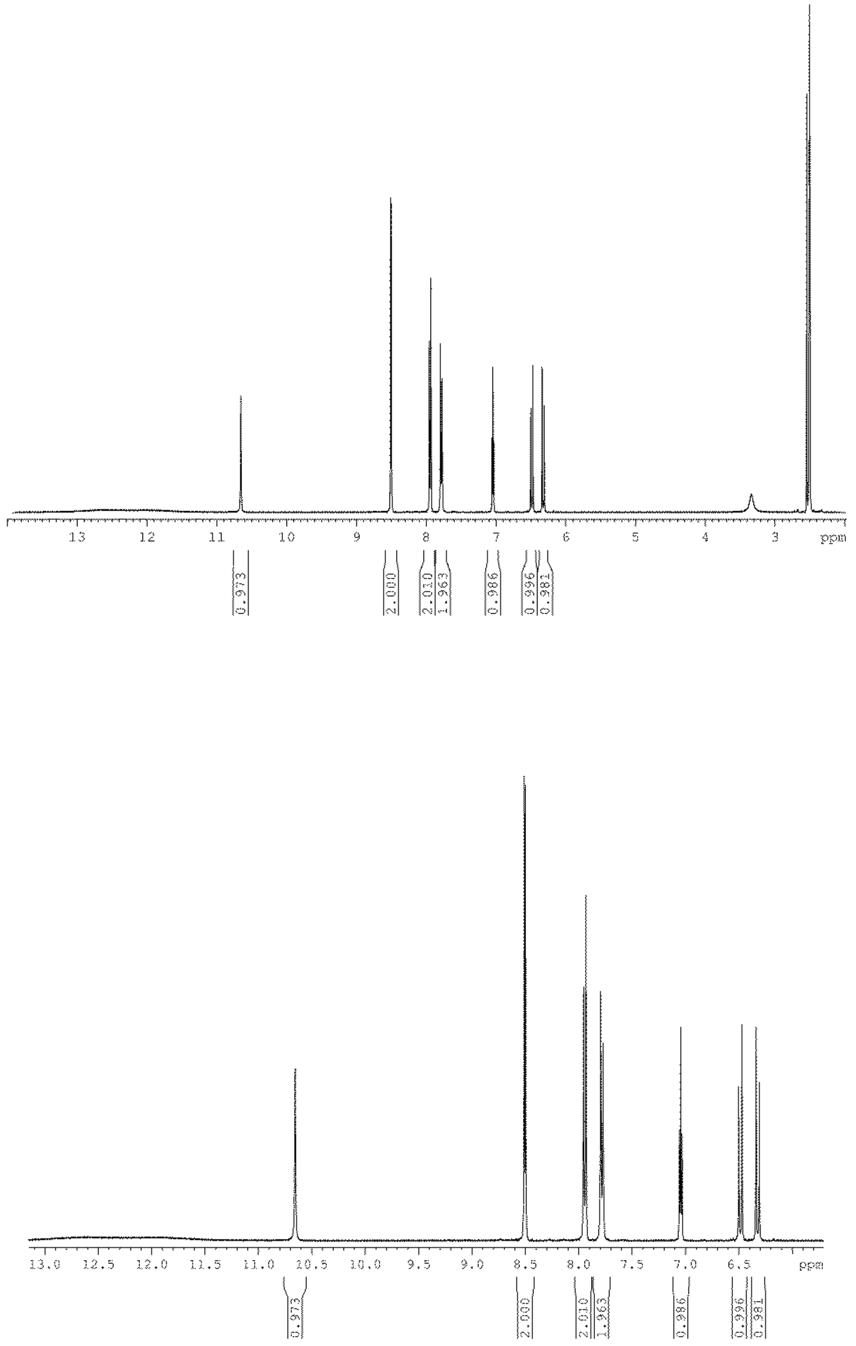

Figure S3. NMR spectra of SDMA (with enlarged zone of characteristic protons).

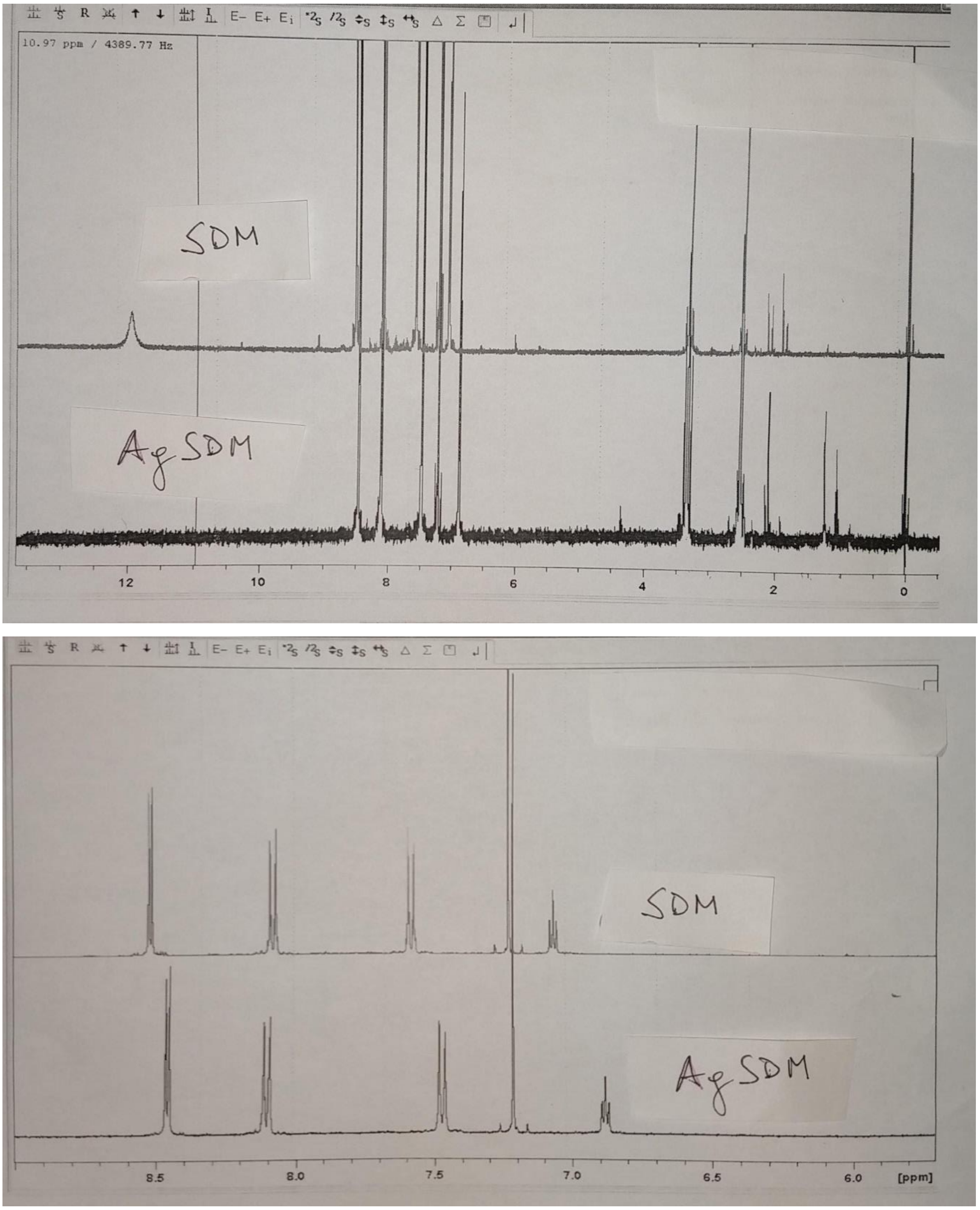

Figure S4. NMR Spectra for SDM and AgSDM (with enlarged zone of characteristic protons).
